# A Live Cell Imaging‐Compatible Bioreactor for the Interrogation of Cellular Responses to Modulated Flow Conditions

**DOI:** 10.1002/advs.202417141

**Published:** 2025-05-11

**Authors:** Subashree Srinivasan, Valerie H. Huhle, Claudia C. Bippes, Nina Tanner, Stephan Frank, Hanspeter E. Killer, Corina Kohler, Albert Neutzner

**Affiliations:** ^1^ Department of Biomedicine University Hospital Basel & University Basel Basel Switzerland; ^2^ Institute of Pathology University Hospital Basel Basel Switzerland; ^3^ Present address: The Francis Crick Institute 1 Midland Road London NW1 1AT UK

**Keywords:** 3D printing, arduino, bioprinting, in vitro model, perfusion cell culture, subarachnoid space

## Abstract

Although being invaluable tools in biomedical research, traditional 2D cell culture models often fail in recapitulating complex environments, limiting their predictive power. To address this limitation, it have developed an open‐source, low‐cost system that combines long‐term 3D cell culture under controlled perfusion conditions with live‐cell imaging. By integrating bioprinted extracellular matrix‐based scaffolds, this study mimics mechanical and biochemical cues experienced by cells within complex tissue contexts. Here, this system is used to generate a model of the cerebrospinal fluid‐filled subarachnoid space to study responses of resident cell types such as meningothelial cells to altered fluid flow conditions. Using fluorescent biosensors, it demonstrates that meningothelial cells respond to modulated fluid flow by differentially activating focal adhesion kinase, a key mechanosensor. The model thus not only provides a powerful platform for investigating the impact of mechanical and other cues on cellular responses, but also bears great potential for the generation of impactful cell biological and pathophysiological models.

## Graphical abstract

An open‐source, low‐cost 3D perfusion system integrating bioprinted scaffolds and live‐imaging overcomes 2D culture limitations. Modeling the cerebrospinal fluid‐filled subarachnoid space, it reveals flow‐dependent focal adhesion kinase activation in meningothelial cells, demonstrating a powerful platform for studying mechanobiology and creating pathophysiological models.

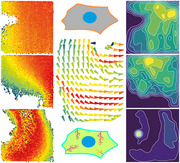

## 1 Introduction

Cell culture‐based models are essential tools for biomedical research. However, given their reductive nature, they are limited with regard to the types of biological questions that can be addressed. To widen the applicability of such models, various adaptations of standard 2D culture systems can be implemented. While coating extracellular matrix (ECM) components onto standard cell culture plates can already provide a more physiological environment, the range of biochemical cues in such models can be further expanded by co‐culturing different cell types. In addition to biochemical signals, cells are also subjected to physical cues such as growth substrate stiffness and mechanical stimulation through pressure and flow. Moreover, switching from 2D to 3D cell culture can further increase the validity of in vitro models. In this context, bioprinted, ECM‐based 3D scaffolds provide cells with a more relevant growth environment regarding stiffness and also geometry. Furthermore, modulating mechanical conditions such as pressure and flow can activate mechanosensitive ion channels or focal adhesion kinases (FAK) and thereby impact cellular behavior.^[^ ^1^ ^]^

Various cell types are subjected to and respond to a wide range of mechanical cues. For example, mechanical stress experienced by osteoclasts and osteoblasts is translated into bone remodeling,^[^ ^2^ ^]^ while cyclic mechanical stimulation of lung epithelial cells affects their secretory activity,^[^ ^3^ ^]^ and fluid motions sensed by hair cells of the inner ear are converted into the perception of sounds.^[^ ^4^ ^]^ Cells directly interfacing with moving fluid such as blood flow‐exposed endothelia or meningothelial cells (MECs) exposed to cerebrospinal fluid (CSF) flow are currently of particular scientific interest. Next to specialized border‐associated macrophages and other immune cells,^[^ ^5^, ^6^ ^]^ MECs are the major cellular component of the meninges.^[^ ^7^ ^]^ Encasing the central nervous system (CNS), the three‐layered meninges are formed by dura, arachnoid, and pia mater. In addition to providing physical protection to the CNS, the meninges are a hub for neuroimmunological processes^[^ ^8^ ^]^ and act as stem cell niche.^[^ ^9^ ^]^ Together, arachnoid and pia mater delimit the subarachnoid space (SAS), a narrow, CSF‐perfused compartment surrounding the CNS. CSF flow through the SAS is involved in brain waste clearance via meningeal lymphatic vessels.^[^ ^10^ ^]^ Of note, given that MECs are powerful phagocytes capable of taking up not only bacteria and apoptotic bodies, but also brain neurotoxic peptides such as amyloid beta and *〈*‐synuclein,^[^ ^11^ ^]^ the large surface of MEC‐CSF interface^[^ ^12^ ^]^ is thought to be absolutely critical for the clearance of toxic metabolites from the brain.^[^ ^11^, ^13^, ^14^, ^15^ ^]^

Here, we engineered a 3D culture system allowing long‐term cell culture as well as live cell imaging under controlled perfusion conditions. Due to its self‐contained nature and custom control software, this system lends itself to parallel operation of multiple instances in standard cell culture incubators. Integrating bioprinted 3D scaffolds, we applied this setup to mimic CSF flow through the SAS to study the interaction between flow and MECs. Using a genetically encoded fluorescent biosensor for FAK activity, we demonstrate how MECs respond to varying CSF flow‐like conditions. In a broader context, our cell culture system might generally prove useful for studying cell types directly exposed to fluid flow. In particular, respective model systems could be employ to study a wide range of diseases including e.g. disorders of the ciliopathy spectrum^[^ ^16^ ^]^ and glymphatic dysfunction‐associated neurodegenerative conditions such as Alzheimer's and Parkinson's disease.^[^ ^17^ ^]^

## 2 Results

**Figure 1:**
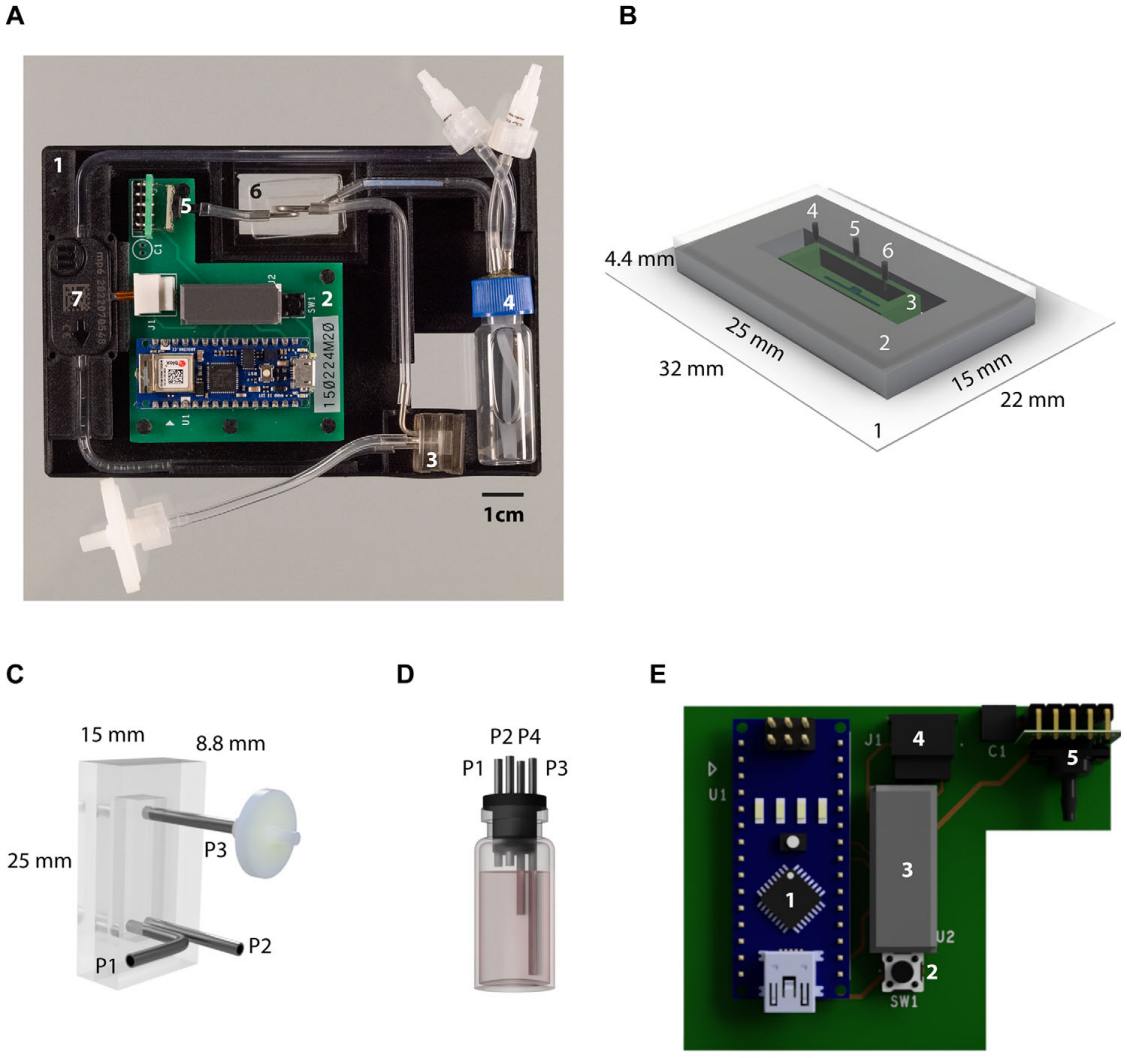
A) Perfusion‐controlled 3D model system on a custom holder (1) manufactured using 3D printing containing a microcontroller mounted on a PCB to regulate pressure and operational parameters (2), custom‐made PDMS bubble trap to remove air bubbles from the media flow (3), a media reservoir (4), a sensor to monitor the pressure within the cell culture system (5), a GelMA scaffold housed in a PDMS chamber (6), and a micropump (7). B) Schematic of a 3D printed GelMA scaffold enclosed in a PDMS chamber. A GelMA scaffold (3) is 3D‐printed onto a standard glass slide (1) and enclosed with a PDMS chamber (2) with inlet (4), pressure sensor port (5), and outlet (6) ports. C) Schematic of bubble trap fabricated from PDMS with media inlet and outlet port (P2 & P1). An air exchange port (P3) capped with a hydrophobic PTFE filter allows for pressure exchange and aids in air bubble removal. Dimensions in B and C are in mm. D) Schematic of a media reservoir with media inlet (P1) and outlet (P4) and media exchange/compound addition port (P3). A PFTE filter capped port (P2) supports air exchange and aids bubble removal. E) Schematic of culture system control board encompassing Arduino Nano IOT 33 microcontroller (1), push switch (2), pump driver IC (3), Molex right angle connector (4) to connect piezoelectric micropump and piezoresistive silicon pressure sensor (5).

**Figure 2:**
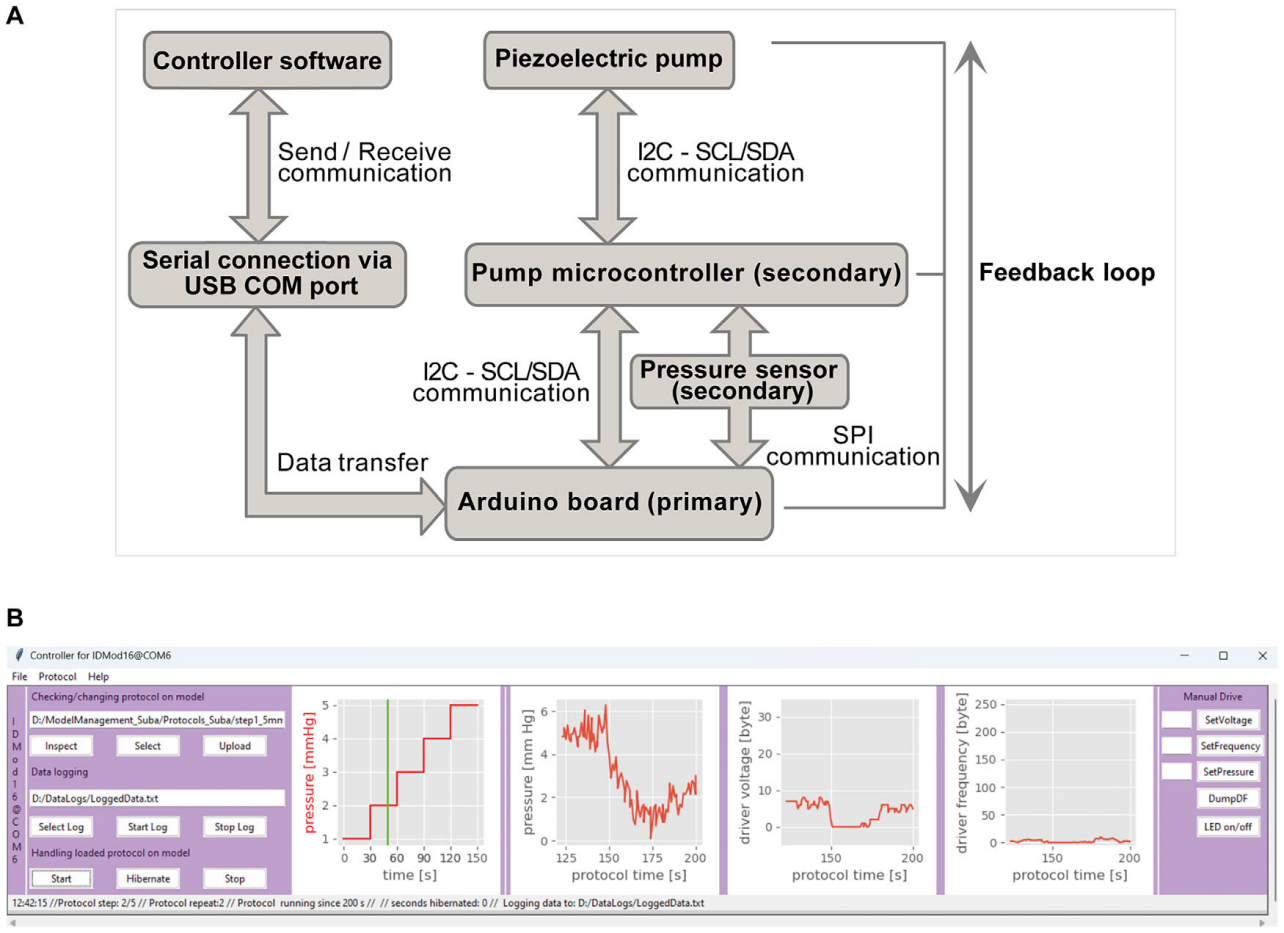
A) Diagram of the hardware‐software architecture of the culture system. The on‐board Arduino microcontroller executes firmware to manage data transfer with the pump microcontroller and pressure sensor through I2C and SPI communication protocols, respectively. Through a serial connection, the microcontroller communicates with graphical user interface software on a connected PC. B) Graphical user interface software written in Python and running on a Windows control PC communicates with the firmware on the microcontroller. The GUI implements functions to design, upload, start, and stop pressure profile protocols, run the system in manual mode, prepare the system for dis‐ and reconnection (“Hibernate”), and monitor as well as log relevant data. Using the GUI, a built‐in LED can be switched to help locate a certain cell culture system among up to twenty systems connected to the same control PC. Both, firmware and control software are available as open source from https://github.com/neutznerlab/bioreactor for non‐commercial use.

For extended cell cultures on 3D‐printed scaffolds under defined flow conditions, we generated a self‐contained perfusion setup the size of a microwell plate compatible with commercially available live cell imaging setups (**Figure** **1**A). To this end, we combined a perfusion chamber (Figure 1B), a media reservoir (Figure 1C), and a bubble trap (Figure 1D), with a piezo‐electric micropump, an integrated circuit (IC) acting as pump driver, a pressure sensor as well as an Arduino microcontroller. We integrated these components on a 3D‐printed holder. The perfusion chamber was designed to encapsulate gelatin‐methacrylate (GelMA) scaffolds, 3D‐printed directly onto coverslips. These scaffolds were enclosed by plasma‐bonding a polydimethylsiloxane (PDMS) housing to the glass surface. To perfuse the scaffold, a piezo‐electric micropump addressable by an IC pump driver was used, allowing for flow rates between 8 µL min^−1^ and 10.000 µL min^−1^. To prevent air bubble entrapment in the perfused scaffold and to allow for quick pressure exchange, a bubble trap manufactured from PDMS was included as was an additional air exchange port in the media reservoir. To maintain aseptic conditions within our setup, these air exchange ports were capped with a hydrophobic polytetrafluoroethylene (PTFE) filter. A sensor directly connected to the perfused culture chamber was incorporated for pressure monitoring. Using a custom printed circuit board (PCB – Figure 1E), we connected pressure sensor, micropump and pump driver with an Arduino‐based microcontroller. On the microcontroller, we implemented firmware in form of a state‐machine (a) to address the pump via the pump driver IC, (b) to readout the pressure sensor, (c) to regulate pump parameters for reaching and maintaining pressure targets, and (d) to execute a multistep protocol for imposing dynamic pressure profiles over time. The firmware implements a simple text‐based protocol to communicate internal parameters and to receive commands from a connected personal computer (PC) running a custom graphical user interface written in Python (**Figure** **2**A,B). This allows simultaneous real‐time monitoring of pressure and pump parameters of more than twenty independent cell culture systems with one PC. The software also facilitates data logging on the connected PC, and allows to generate and upload protocols for imposing pressure profiles on the culture chamber. Moreover, it permits placing the firmware into a “hibernation state” to transparently dis‐/re‐connect from the PC or external USB power sources without disrupting currently executed pressure profile protocols. Thus, our setup can be disconnected from its power source for media exchange or live cell imaging outside of cell culture incubators while returning to previous running conditions upon restoration of power. As for power sources, the culture system can be operated on a standard USB port, a powered PC‐connected USB hub, or for portability on a USB power bank lacking low power detection.

**Figure 3:**
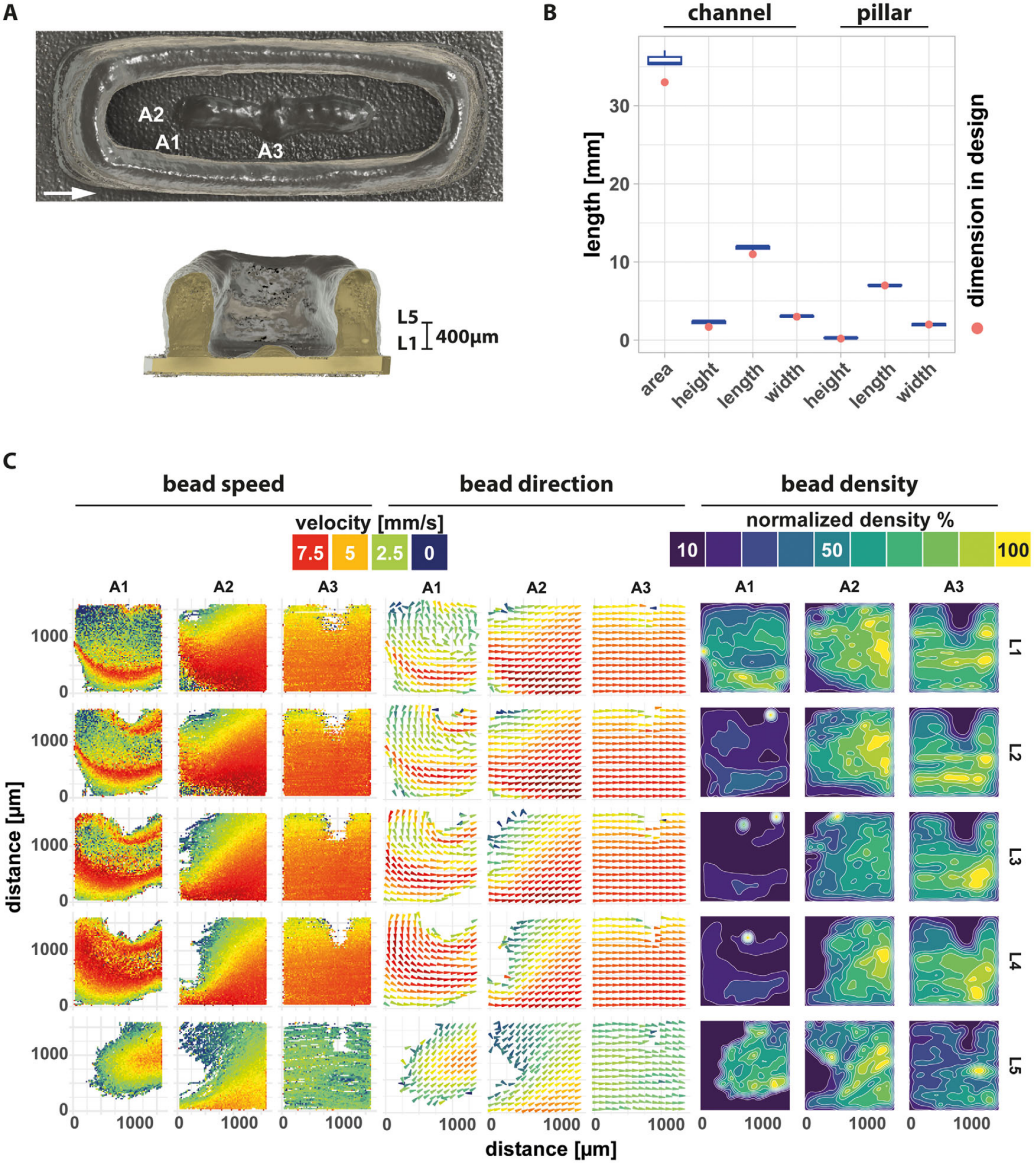
A) Top‐view and cross‐sectional micro‐CT images of a bioprinted 3D GelMA scaffold. The arrow indicates flow direction and labels A1 to A3 and L1 and L5 indicate imaging positions as used in C. B) Dimensions of 3D‐printed GelMA scaffold (n = 3) where determined from micro‐CT images and difference between printed scaffold and CAD model was determined. C) Red‐fluorescent latex beads (Ø 1 µm) were perfused at 1 mmHg through a 3D‐printed GelMA scaffold as shown in (A) and imaged using confocal microscopy at three different locations (A1‐A3) in five focal planes (L1‐L5). Bead movement between two consecutive frames was tracked to measure bead speed and direction. Plotted are velocity of individual beads (left panel), averaged speed and direction (middle panel) and normalized bead density (right panel).

Our culture system is designed for the perfusion of scaffolds with maximal dimensions of 5 mm × 15 mm × 2.7 mm. To assess the suitability of the perfusion setup, we 3D‐printed a GelMA scaffold containing two channels separated by a pillar with two obstacles extending into the channels. When imaged by micro‐computed tomography (**Figure** **3**A), 3D‐printed scaffolds were found to closely follow specifications with only minor inter‐scaffold deviation (Figure 3B). To measure local flow rates, fluorescent latex beads perfused across a GelMA scaffold were imaged. Using particle detection and tracking, we obtained speed, flow direction, and density maps for a GelMA scaffold. We measured particle speeds between 0 and 8.9 mm s^−1^ and found scaffold geometry to greatly influence bead velocity. As expected, obstacles in the channel resulted in lower speed and turbulent behavior, which in a cell culture setting leads to location‐dependent physical cues to cells at different scaffold positions (Figure 3C).

**Figure 4:**
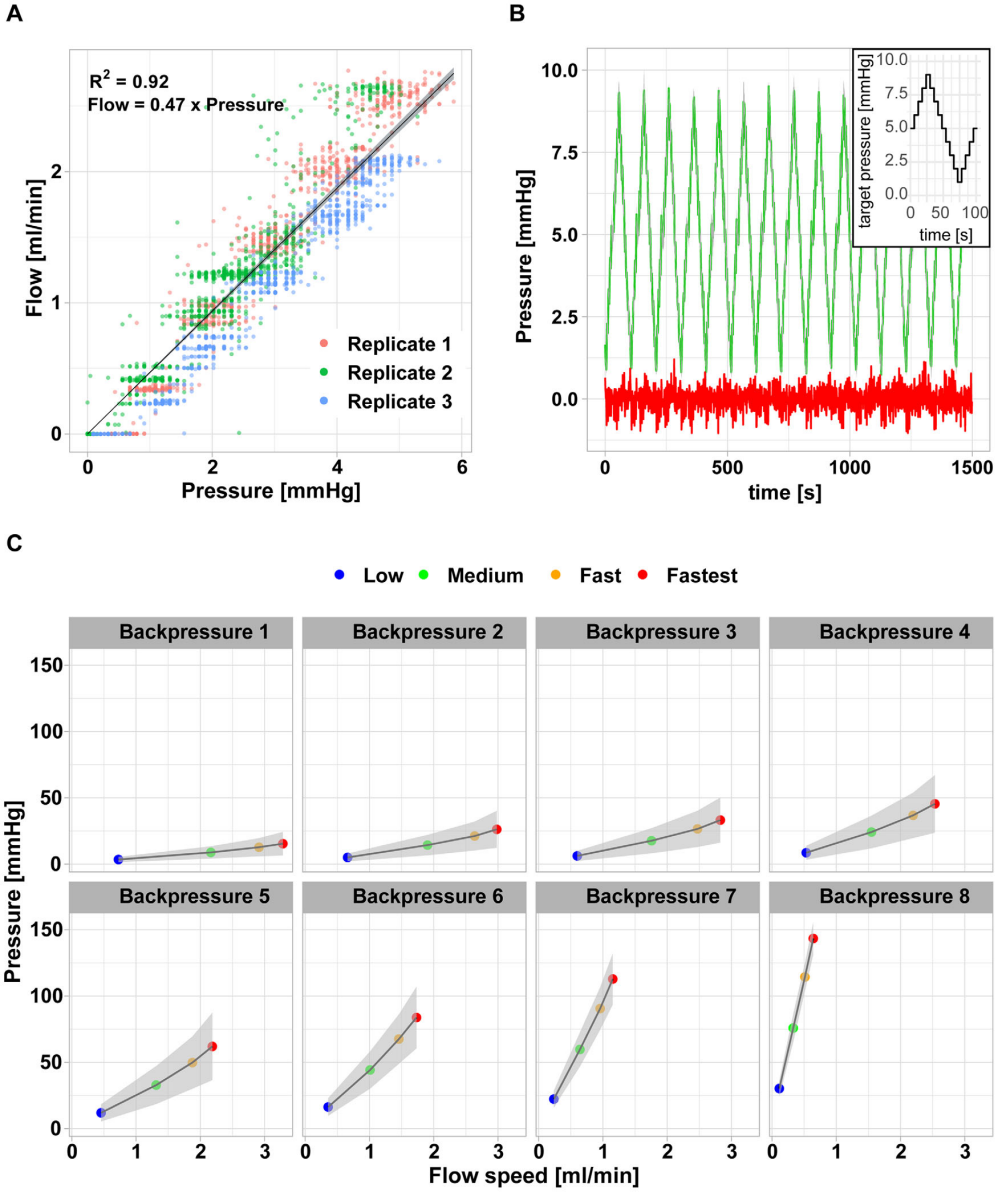
A) By imposing a target pressure on culture setups (n = 3) and measuring the resulting flow, a pressure‐flow relationship was established. Linear regression was performed (R^2^ = 0.92) revealing a linear relationship between pressure and flow (flow = 0.47×pressure). B) An oscillatory target pressure profile (inset) was imposed on cell culture systems (n = 3) and actual pressure was measured (green line). The difference target – actual pressure is plotted in red. C) A four‐step target pressure protocol was imposed on culture systems (n = 3) modified to allow for increasing backpressure by restricting media outflow at the chamber outlet. Shown is the flow‐pressure relationship (grey ribbon: pressure and flow standard deviations).

Given the size constraints of live imaging setups, we opted to rely solely on a pressure sensor for flow monitoring and refrained from integrating a flow sensor into the setup. To obtain a pressure‐flow rate relationship, an external flow sensor was connected to the setup. Upon repeatedly measuring flow rates in relation to target pressures, we found flow rate and pressure to be linearly related with relationships being similar between individual models (**Figure** **4**A). Thus, flow rates can be estimated based on pressure monitoring for a given culture system configuration.

In general, cells exposed to flow are subjected to fluctuations in flow speed at different time scales, for example, as a consequence of heartbeat, breathing rhythm, or movement. To simulate these conditions in our culture system, we assessed the dynamic range of pressure regulation (Figure 4B). To this end, we applied a target pressure protocol by approximating a sinusoidal wave trough to peak from 1 to 9 mmHg over 120 s (inset Figure 4B), and found culture chamber pressure to react almost instantaneously to changes in pump speed over the target pressure range for extended periods of time (Figure 4B). Next, we measured the pressure profile in perfusion chambers resulting from different flow rates in relation to different system backpressures (Figure 4C). Flow rates between 0.1 ± 0.1 and 3.3 ± 0.6 mL min^−1^ and intra‐chamber pressures ranging from 3.5 ± 2 to 143.3 ± 12.1 mmHg were achieved before either maximum pump settings were reached or media leakage occurred.

Next, we assessed the suitability of the perfusion system for extended cell culture. For this, two cell types from two different perfused environments were grown on 3D‐printed GelMA scaffolds. First, HMEC‐1 cells^[^ ^18^ ^]^ representative for vascular endothelium exposed to blood flow were grown for 2 weeks at a pressure of 25 mmHg and a flow rate of about 1.5 mL min^−1^ using backpressure restriction in the outflow pathway to mimic mean flow speed in arterioles and venules.^[^ ^19^ ^]^ As shown in Figure S1 (Supporting Information), HMEC‐1 populated the GelMA scaffold and were imaged by confocal microscopy.

**Figure 5:**
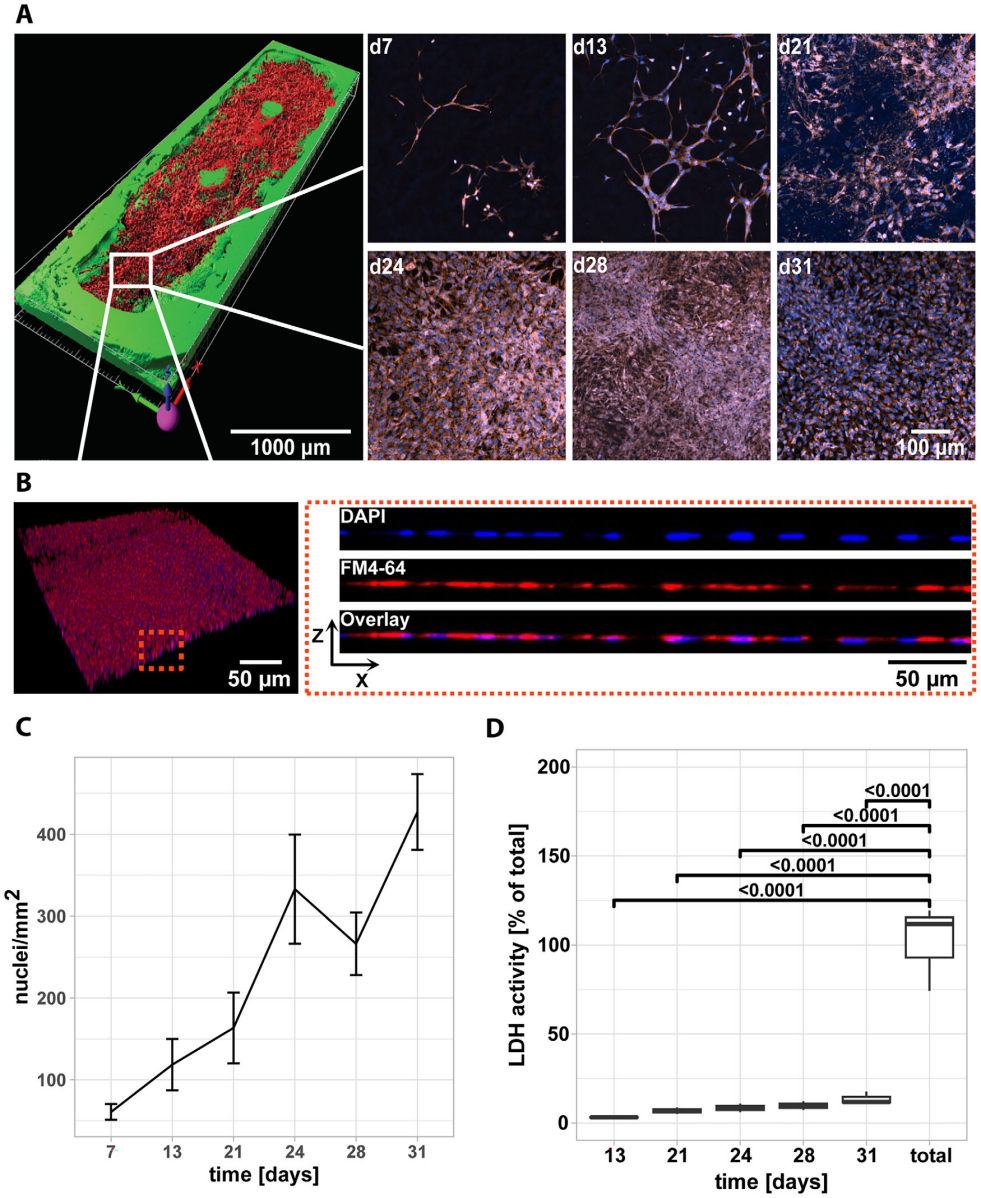
A) Ben‐Men‐I cells grown for 7, 13, 21, 24, 28, and 31 days on 3D‐printed GelMA scaffolds (n = 4) were stained to reveal nuclei (DAPI – blue) and plasma membrane (FM4‐64 – red) and imaged using confocal microscopy. The 3D reconstruction shows a representative scaffold after 31 days of cultivation. B) Shown is a 3D reconstruction of Ben‐Men‐I cells grown 31 days on GelMA scaffold and stained as in A. C) Nuclei were counted using image analysis to determine cell density on scaffolds (n = 4). D) Ben‐Men‐I cells were cultivated on GelMA scaffolds (n = 3), media was sampled at indicated time points before media exchange, and lactate dehydrogenase (LDH) activity was measured. LDH activity is expressed as a percentage of total LDH activity determined through endpoint cell lysis. Statistical significance was assessed using ANOVA and Tukey post‐hoc test.

Next, using Ben‐Men‐I cells as model for MECs,^[^ ^20^ ^]^ we established a perfused meningeal cell culture model. MECs lining the SAS are exposed to flowing CSF and pressures between 0 and 15 mmHg corresponding to physiological intracranial pressure.^[^ ^21^ ^]^ Upon seeding, Ben‐Men‐I cells populated GelMA scaffolds and formed a dense monolayer (**Figure** **5**A,B). Quantitative image analysis revealed increasing cell density over time with 61 ± 10 cells mm^−2^ on day 7 to 427 ± 46 cells mm^−2^ on day 31 after seeding (Figure 5C). To test for cell death of Ben‐Men‐I grown on perfused GelMA scaffolds for up to 31 days, we repeatedly measured lactate dehydrogenase (LDH) levels in the perfusate prior to media exchange. Consistent with rising cell numbers over time, we found increasing LDH concentrations in the perfusate ranging from 3.2 ± 0.3% on day 13 to 13.7 ± 3.5% on day 31 with percentages relative to complete LDH release at end of experiment (Figure 5D). These data are consistent with a 4.5% daily loss of cells as media was exchanged every third day.

**Figure 6:**
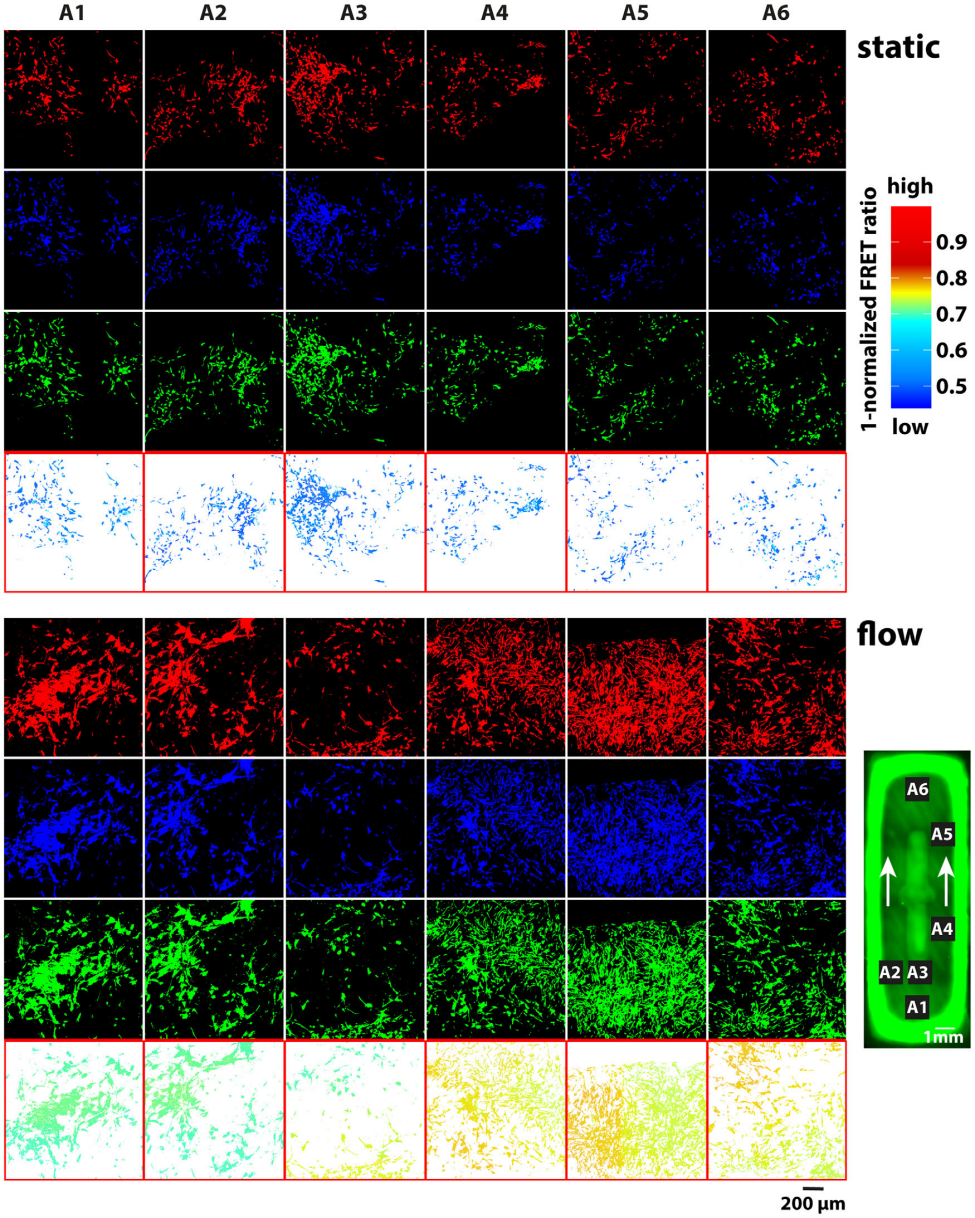
Influence of flow dynamic on FAK activity of MECs. Ben‐Men‐I cells co‐expressing red‐fluorescent tdTomato protein and blue‐green FRET biosensor grown on GelMA scaffolds under static (upper panel) or flow conditions (lower panel) were imaged at six different scaffold locations by confocal microscopy (A1‐6 – arrows indicate flow direction, number of scaffolds ≥3). Cells were segmented based on the red channel signal using Otsu thresholding. The FRET ratio indicative of FAK activity was calculated as signal blue channel/signal green channel and normalized to 1. FAK activity is expressed as 1‐FRET ratio with higher values indicating higher FAK activity.

Genetically encoded fluorescent protein biosensors allow for the non‐destructive measurement of cellular parameters. Here, we assessed the mechanosensitivity of Ben‐Men‐I cells stably expressing a Förster resonance energy transfer (FRET)‐based FAK biosensor. Cultured on GelMA scaffolds under static or flow conditions for 14 days, cells were imaged by confocal microscopy. Cells cultured on GelMA support in the absence of media flow showed uniformly low FAK activity (**Figure** **6** – upper panel). Interestingly, when cultured on standard 2D cell culture substrate, FAK activity was still uniform across different locations and in between cells (Figure S2, Supporting Information); however, FAK activity was elevated compared to cells cultured on non‐perfused GelMA substrates (Figure 6). When analyzing cells cultivated at a flow speed of ≈1 mL min^−1^ corresponding to 2 mmHg pressure, we found location‐dependent FAK activation reflecting the different local flow speeds within the geometrically complex scaffold. While FAK activity was higher near the media entry, consistent with higher velocity in this area (Figure 6 – location A1, also compare to Figure 3C), gradually decreasing FAK activity was observed in the scaffold channels consistent with lower flow speed at these scaffold locations.

## 3 Discussion

We generated an open‐source, low‐cost system for the longer‐term culture of cells on 3D scaffolds under varying flow conditions. Integrating bioprinted 3D scaffolds that mimic ECM and tissue geometry with mini‐fluidics to precisely control physical parameters such as pressure and flow rates over wide ranges, our system also allows for live cell imaging and non‐invasive measurement of cellular parameters using genetically encoded fluorescent biosensors. Extensive live‐cell imaging techniques enable comprehensive readouts, while the companion software provides a user‐friendly interface for real‐time system monitoring, data logging, automated flow‐pressure regulation, and transparent (re‐)connection of culture systems to the control PC. Thus, our setup enables the generation of noninvasive, yet accessible, cellular models mimicking physiological conditions encountered by cells in vivo.

The cell culture system was designed with the space constraints of live cell imaging setups as well as unit costs in mind. In contrast to most peristaltic pumps, its piezo‐electric pump is more compact with favorable clearance and a wide flow speed range. For monitoring the culture system, we included a relative pressure sensor directly connected to the perfusion chamber. We opted against incorporation of flow sensors due to their relatively large size and the high costs of sensors with suitable measurement windows. With a unit cost of about 200 USD, its compact size, almost complete reusability, and the possibility to share the controlling PC between more than twenty systems, our setup is perfectly suited for parallel operation. 3D‐printed racks (Figure S3, Supporting Information) allow stacking of culture systems and their organization inside a cell culture incubator, thus efficiently utilizing space and resources. While presented here for perfusion of 3D‐printed scaffolds, the culture system might also be useful in the absence of a dedicated bioprinter. Biocompatible 3D scaffolds can also be molded,^[^ ^22^ ^]^ or glass slides can be coated with ECM components for perfused 2D cell culture.

Highlighting the versatility of our system, we successfully cultivated two cell types that would physiologically interface either with blood or CSF. Employing this system, we generated a simplified model of the SAS by cultivating MECs on a collagen‐based, 3D‐printed ECM. Constantly in contact with flowing CSF, we hypothesized that MECs are mechanosensitive cells capable of dynamically reacting to physical stimuli such as varying levels of shear stress caused by changes in CSF flow velocity. Interestingly, in optic nerve sheath compartment syndrome, a condition connected to cases of papilledema and normal tension glaucoma, the SAS of the optic nerve is no longer normally perfused with CSF.^[^ ^23^ ^]^ Here, MECs seem to react to altered flow conditions as reflected by upregulated L‐PGDS levels,^[^ ^24^ ^]^ an important soluble transport protein of the CSF produced by MECs. And indeed, when altered flow conditions were applied to our SAS model, MECs reacted to flow rate changes via FAK activation. In fact, FAK activity patterns were found to closely resemble fluid velocity distribution across different scaffold regions. Collectively, these observations confirm that MECs are mechanosensitive and capable of sensing differences in flow pressure and velocity. As MECs functionally respond to pathological flow conditions in humans, our findings also support a pathophysiological role for these cells in optic nerve and brain diseases such as normal tension glaucoma and Alzheimer disease, respectively. By leveraging off‐the‐shelf electronic components and 3D‐printing we arrived at a versatile, low‐cost perfusion setup for generating in vitro cellular models amenable to direct observation with genetically encoded biosensors allowing to address complex biological questions. Thus, our system proved useful to address questions of importance in the field of CSF flow related to glymphatic dysfunction‐associated neurodegenerative conditions including Alzheimer's and Parkinson's disease.^[^ ^17^ ^]^ Beyond the subarachnoid space, seen in a broader medical context, our culture system could prove particularly impactful for studying ciliopathy spectrum disorders^[^ ^16^ ^]^ where primary cilia are dysfunctional or absent. As a major cellular mechanosensor, primary cilia provide developmental clues and help to maintain cell polarity. This is exemplified by situs inversus^[^ ^25^ ^]^ or polycystic kidney disease^[^ ^26^ ^]^ where dysregulated flow sensing causes left‐right mirrored visceral organ disarrangement or kidney damage due to dysregulated epithelial cell growth, respectively.

## 4 Experimental Section

### Scaffold Printing and Assembly of Cell Culture Perfusion Chamber

For bioprinting of 3D scaffolds, 0.5 mg of lyophilized GelMA and 0.1 mg of photo‐initiator irgacure (CELLINK, VL3500000502) were dissolved in 10 mL of PBS (Sigma‐Aldrich, D8537) at 50 °C for 1 hour. The prepared GelMA/irgacure mix was sterile filtered (Carl Roth AG, KH54.1) and filled into a disposable syringe (Nordson EFD, 7366092) for extrusion printing with a 3D Discovery bioprinter (RegenHU, Switzerland). GelMA was extruded at 24 °C onto a 22×32 mm cover glass (Menzel‐Gläser, BB022032A1) mounted onto a Peltier element (KIMILAR, 521575826845) kept at 5 °C with an extrusion pressure between 0.036 and 0.066 MPa and a feed rate between 9 and 12 mm s^−1^. After printing, the scaffold was exposed to UV light (365 nm) for 5 min to cross‐link and achieve structural integrity. The scaffold was sealed using a PDMS housing. The cover glass with scaffold and PDMS housing was exposed for 60 s to oxygen plasma (Harrick Plasma, PDC‐002‐CE) and subsequently bonded by applying gentle pressure to obtain a leak‐free seal. Access holes were punched into the PDMS using 1.5 mm Rapid‐Core Microfluidic punches (Darwin microfluidics, PT‐T983‐15) for an inlet port and 1.2 mm punches for outlet and pressure sensor port (Darwin microfluidics, PT‐T983‐12). PDMS connectors (Darwin microfluidics, Outlet – PN‐BEN‐16G‐20, Inlet & pressure access port PN‐BEN‐18G‐20) were inserted into the punched holes.

### Manufacturing of Perfusion Cell Culture System

A 96 well plate‐sized holder (83×125 mm) was printed from tough polylactic acid (PLA, Ultimaker, 8850261) on an Ultimaker S3 3D printer after preparing a G code file using CURA slicing software (Ultimaker, Version 5.0.0). An Arduino Nano 33 IoT (Arduino, ABX00032), a mp‐high driver IC (Bartels Mikrotechnik GmbH, BM‐E‐0002), a right‐angled molex connector (Molex, 39532044), and a pressure sensor (Honeywell, ABPDANV015PGSA3) were connected via a custom PCB (JCLPCB). The PCB was placed onto the holder and secured by tight‐fitting integrated PLA columns. A piezo electric micropump (Bartels Mikrotechnik GmbH, BM‐P‐0008) was connected to the PCB via the molex connector. A media reservoir was made from a 4 mL glass vial (Infochroma AG, G074B‐14/045‐SKFW16‐H) by inserting four metal couplers through the included rubber septum. Two couplers serve as media inlet (Darwin Microfluidics, PN‐STN‐16G‐20) and outlet (Darwin Microfluidics, PN‐STN‐18G‐20), respectively. Two additional couplers (Darwin Microfluidics, PN‐STN‐16G‐20) terminated with PTFE hydrophobic filters (Sigma–Aldrich, SLFGR04NL) permit air exchange. A bubble trap was made from PDMS by plasma‐bonding two PDMS housings and inserting two metal couplers as media in‐ and outlets and one metal coupler terminated with a hydrophobic filter (Carl Roth, P817.1) as an air exchange port. An oxygen‐permeable silicone tube (Bartels, #mp‐t ID1.3) with a diameter of 1.3 mm was connected from the reservoir to a piezoelectric pump, which was then linked to a bubble trap, followed by a perfusion chamber, a pressure sensor, and finally routed back to the reservoir. A smaller diameter tubing (1.02 mm, Saint‐Gobain Tygon LMT‐55 ID) served as perfusion chamber outlet. To adjust backpressure, this outlet tubing either contained another small tube (Microfluidics, Darwin microfluidics, SKU: DM‐PTFE‐0805‐20) or was squeezed using an adjustable screw. To prepare for aseptic operation, the cell culture system was rinsed for 5 min each with 4 mL of sterile, deionized water, followed by 70% ethanol, sterile deionized water, PBS, and finally cell culture media. Assembled culture systems maintained aseptic conditions for over 1 month.

### Cell Culture

Ben‐Men‐1 cells were cultured in Dulbecco's Modified Eagle Medium (DMEM, Sigma‐Aldrich, D5671) supplemented with 10% FBS (Sigma–Aldrich, F7524), 1 mM Sodium Pyruvate (Sigma–Aldrich, S8636), 2 mM L‐glutamine (Sigma–Aldrich, 59202C), and 1:100 penicillin‐streptomycin solution (Sigma–Aldrich, P4333) in a humidified incubator at 37 °C with 5% CO₂. For cultivation on GelMA scaffolds, cells were harvested by trypsinization (Sigma–Aldrich, T3924), resuspended at 3e5 cells/500 µL, and injected into the cell culture system via the bubble trap. Cells were to settle for 3 days to attach onto the scaffold before starting perfusion.

### Flow Velocity Measurement in Cell Culture System

A flow sensor (EK‐SLF3S‐1300F, Sensirion) was connected between the bubble trap and the inlet of the GelMA channel to measure the incoming flow rate into the perfusion chamber. Vendor provided software was used to obtain time stamped data. Using the data logging function of our control software, time‐stamped pressure data was obtained. Both data sets were synchronized using these time stamps and combined for further analysis.

### Molecular Cloning

To generate a bi‐cistronic 3rd generation lentiviral vector (pUTLRA‐tdTomato‐FAT‐FAK) for expression of a FAK biosensor and red‐fluorescent tdTomato protein, pULTRA‐Chili (Addgene plasmid #48687) was cut with *Xba*I and *Eco*RI‐HF (New England Biolabs) and annealed oligonucleotides 5’‐CTAGATTGGATCCGCTAGCG‐3' and 5’‐AATTCGCTAGCGGATCCAAT‐3' were inserted to allow for subsequent cloning of the FAT FAK gene (Addgene plasmid #78303,^[^ ^27^ ^]^) with *Bam*HI‐HF and *Eco*RI‐HF.

### Lentivirus Production and Transduction

For the production of lentiviral particles, 4e6 HEK293T cells were seeded into a T75 cell culture flask one day prior to transfection. Using 24 µL of JetPEI (Polyplus), 4 µg pULTRA‐tdTomato‐FAT FAK biosensor, 2 µg pMD2.G (Addgene plasmid # 12259,^[^ ^28^ ^]^), and 2 µg pCMVR8.74 (Addgene plasmid #22036,^[^ ^29^ ^]^), cells were co‐transfected according to the manufacturer`s instructions. The next day, cell culture medium was exchanged. After an additional 48 h, cell culture supernatant with viral particles was collected, filtered through a 0.45 µm filter (TPP, 99745) and stored in aliquots at −80 °C until use. Ben‐Men‐1 cells at a confluency of 90% were transduced for 24 h with pULTRA‐tdTomato‐FAT FAK lentiviral supernatant in T25 cell culture flask using a mixture of 2 mL viral particles and 2 mL cell culture medium.

### Microscopy and Image Analysis

Cell culture systems were mounted on a Nikon spinning disk confocal CSU‐W1 microscope and observed using a Photometrics Prime 95B camera. CFI Plan Apo Lambda objective with 10x magnification of numerical aperture 0.45 was used to scan the samples resulting in a pixel size of 1.1 µm. Standard cell culture incubator settings were utilized in the live cell incubation unit. Image acquisitions were controlled using Nikon NIS Elements 4.21 software. To assess cell growth on GelMA scaffolds, cells were fixed with 4% PFA and stained with DAPI and FM4‐64 (Sigma–Aldrich, SCT127) to mark nuclei and plasma membrane, respectively. Scaffolds were imaged by taking advantage of their green autofluorescence. Cell density was determined by counting DAPI‐positive nuclei using the ImageJ Stardist plugin.^[^ ^30^ ^]^ FM4‐64 plasma membrane staining was used to determine cell coverage. To measure FAK activity, confocal images of Ben‐Men‐I cells expressing red‐fluorescent tdTomato and FAK biosensor grown on perfused GelMA were segmented based on tdTomato expression and FRET ratio was determined by dividing fluorescence in the blue by fluorescence in the green channel. To measure flow speed, red fluorescent latex beads with a diameter of 1 µm (Sigma‐Aldrich, #L2778) were perfused through the scaffold at a flow rate of 0.4 mL min^−1^ and imaged at three different locations and five focal planes at a rate of ≈150 images/s for ≈180 s. Using Python packages nd2 (https://pypi.org/project/nd2/) to access confocal imaging files and cv2 to use the image processing library OpenCV,^[^ ^31^ ^]^ location and movement of beads were extracted using blob detection combined with morphological filtering and the Lucas‐Kanade algorithm as implemented in OpenCV.

### Computer Tomography

Micro‐CT scanning of freshly printed GelMA scaffolds was performed using a SkyScan 1275 scanner (S/N: 15B15007, Bruker) equipped with a HAMAMATSU L11871‐20 X‐ray source and a DEXELA 1512 camera.

### Measurement of LDH Activity

To measure LDH release, media perfusate of cells grown on 3D scaffolds was collected before media exchange and analyzed with CyQUANT LDH (ThermoFisher, C20300) according to manufacturer's recommendation.

### Access to Software and Construction Files

The firmware to run on the Arduino Nano 33 IOT used in the cell culture system as well as the source code for the graphical user interface software can be found on GitHub (https://github.com/neutznerlab/bioreactor). STL files for 3D printing of scaffolds and culture system holders, Gerber file for PCB manufacturing as well as a Bill of Materials for the culture system are also found in our GitHub repository.

## Conflict of Interest

The authors declare no conflict of interest.

## Supporting information



Supporting Information

## Data Availability

The data that support the findings of this study are available from the corresponding author upon reasonable request.
