# Supplementary material for: A Live Cell Imaging‐Compatible Bioreactor for the Interrogation of Cellular Responses to Modulated Flow Conditions
Source: Adv Sci (Weinh). 2025 May 11;12(22):2417141. doi: 10.1002/advs.202417141 (PMC12165081; doi:10.1002/advs.202417141)
Supplement: Supplementary file 1 — Supporting Information [file ADVS-12-2417141-s001.pdf]

ADVANCED SCIENCE
Open Access

Supporting Information

for *Adv. Sci.*, DOI 10.1002/advs.202417141

A Live Cell Imaging-Compatible Bioreactor for the Interrogation of Cellular Responses to Modulated Flow Conditions

*Subashree Srinivasan, Valerie H. Huhle, Claudia C. Bippes, Nina Tanner, Stephan Frank, Hanspeter E. Killer, Corina Kohler and Albert Neutzner**

A live cell imaging-compatible bioreactor for the interrogation of cellular responses to modulated flow conditions

Subashree Srinivasan[1], Valerie H. Huhle[1,*], Claudia C. Bippes[1], Nina Tanner[1], Stephan Frank[2], Hanspeter E. Killer[1], Corina Kohler[1,3], Albert Neutzner[1,§]

[*]Contributed equally to this work

[1]Department of Biomedicine, University Hospital Basel & University Basel, Basel, Switzerland

[2]Institute of Pathology, University Hospital Basel, Basel, Switzerland

[3]current address: The Francis Crick Institute, 1 Midland Road, London NW1 1AT, UK

[§]Corresponding author: Albert Neutzner, University Hospital Basel, Department of Biomedicine, Hebelstrasse 20, 4031 Basel, Switzerland. E-mail: albert.neutzner@unibas.ch

Supplemental Material and Methods

Endothelial-like HMEC-1 cells (ATTC, CRL-3243) were cultured in MCDB131 media (Life Technologies, 10372019) supplemented with 10 ng/ml epidermal growth factor (Thermofisher, PHG0314), 1 µg/ml hydrocortisone (Sigma-Aldrich, H0396), 10 mM L-glutamine (Sigma-Aldrich, 59202C), 1:100 penicillin-streptomycin solution (Sigma-Aldrich, P4333) and 10% FBS (Sigma-Aldrich, F7524).

Supplemental Figures

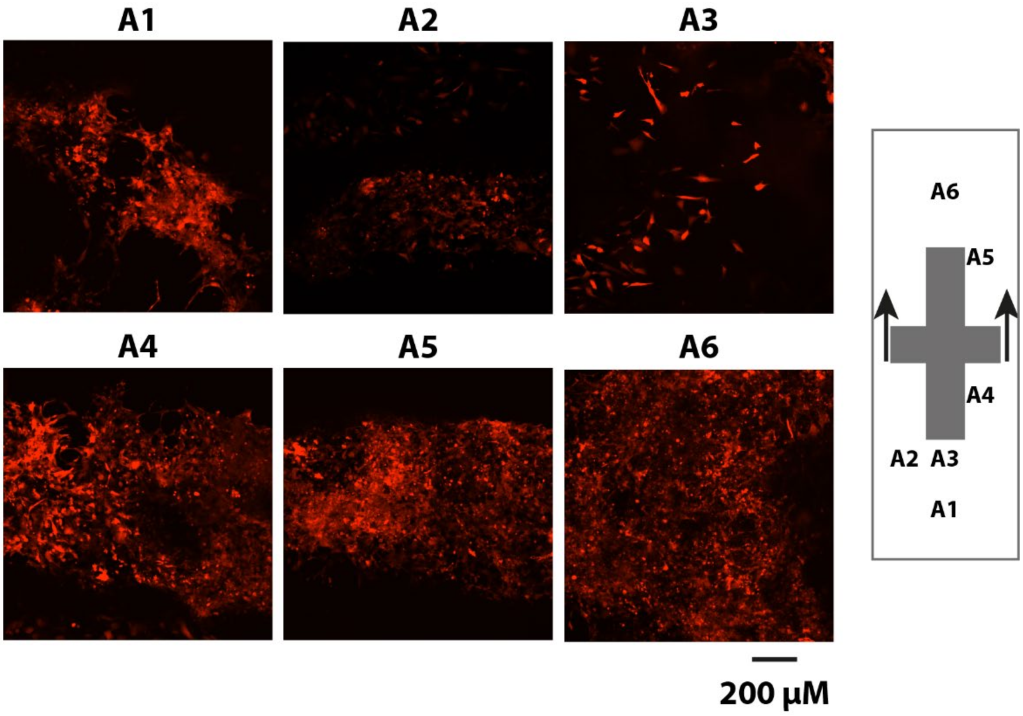

Figure S1 Endothelial-like HMEC-1 cells expressing red-fluorescent tdTomato protein were seeded onto 3D-printed GelMA scaffolds and cultivated for 14 days under 25 mmHg pressure with backpressure set to restrict flow speed to around 1.5 ml/min. Scaffolds were imaged at six different locations as depicted in the schematic. Shown is one representative example out of five samples.

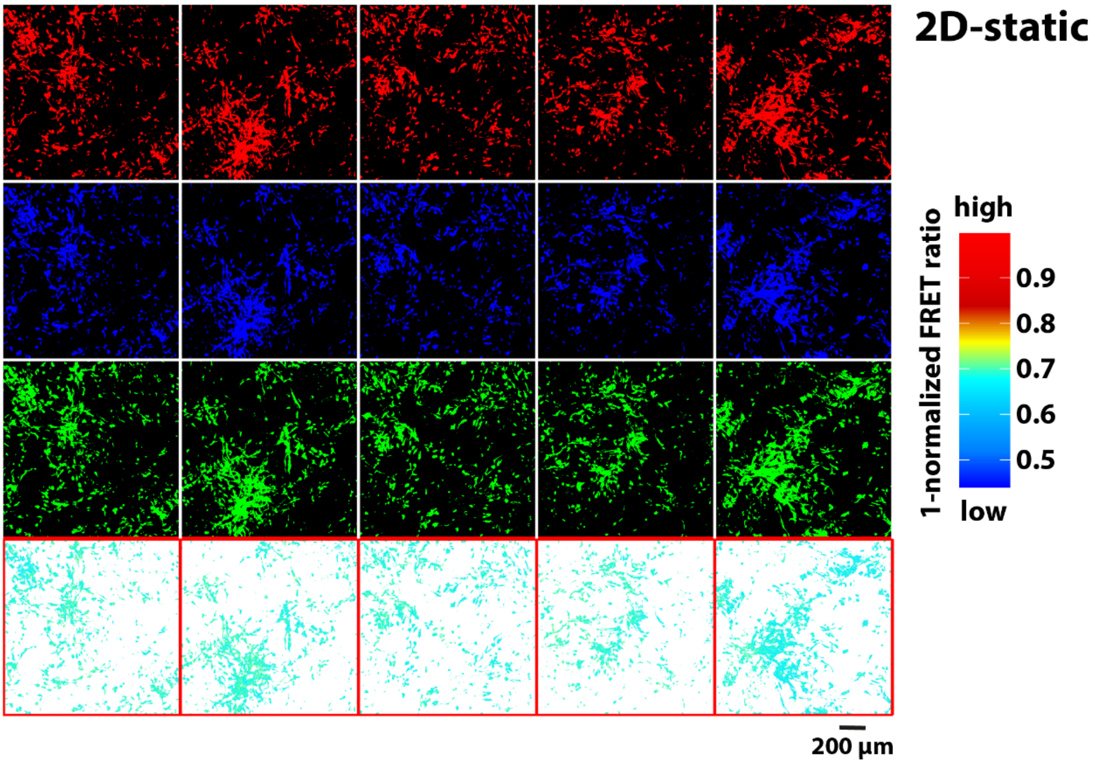

Figure S2 FAK activity in MECs under static conditions on 2D cell culture substrate. Ben-Men-I cells co-expressing red-fluorescent tdTomato protein and blue-green FRET biosensor grown on standard cell culture plasticware were imaged at five different locations using confocal microscopy. Cells were segmented based on the red channel signal using Otsu thresholding. The FRET ratio indicative of FAK activity was calculated as signal blue channel/signal green channel and normalized to 1. FAK activity is expressed as 1-FRET ratio with higher values indicating higher FAK activity. Please note, the color scale used is identical to the scale used in Figure 6 (Ben-Men-I on static or perfused GelMA scaffolds).

A B

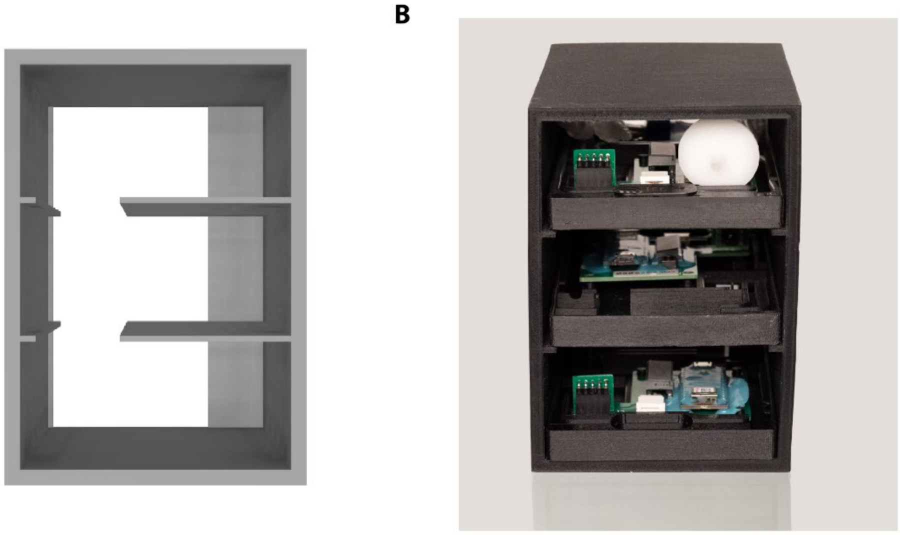

Figure S3 Multi-level rack for cell culture system. **A)** 3D rendering of a CAD model and **(B)** image for a multi-level rack system designed to hold three culture systems per rack. The back panel is designed to organize incoming USB cables connected to each system, ensuring tidy cable management. Please note, Arduino microcontrollers were covered in green Dublisil 15 Speed (Dreve Dentamid GmbH, Germany) to prevent corrosion in the humid atmosphere of the cell culture incubator.
